# Single-Character-Based Embedding Feature Aggregation Using Cross-Attention for Scene Text Super-Resolution

**DOI:** 10.3390/s25072228

**Published:** 2025-04-02

**Authors:** Meng Wang, Qianqian Li, Haipeng Liu

**Affiliations:** School of Information Engineering and Automation, Kunming University of Science and Technology, Kunming 650500, China; liqianqian@stu.kust.edu.cn (Q.L.); ran@kust.edu.cn (H.L.)

**Keywords:** scene text image super-resolution, cross-attention, cross-fertilization, text recognition

## Abstract

In textual vision scenarios, super-resolution aims to enhance textual quality and readability to facilitate downstream tasks. However, the ambiguity of character regions in complex backgrounds remains challenging to mitigate, particularly the interference between tightly connected characters. In this paper, we propose single-character-based embedding feature aggregation using cross-attention for scene text super-resolution (SCE-STISR) to solve this problem. Firstly, a dynamic feature extraction mechanism is employed to adaptively capture shallow features by dynamically adjusting multi-scale feature weights based on spatial representations. During text–image interactions, a dual-level cross-attention mechanism is introduced to comprehensively aggregate the cropped single-character features with textual prior, also aligning semantic sequences and visual features. Finally, an adaptive normalized color correction operation is applied to mitigate color distortion caused by background interference. In TextZoom benchmarking, the text recognition accuracies of different recognizers are 53.6%, 60.9%, and 64.5%, which are improved by 0.9–1.4% over the baseline TATT, achieving an optimal SSIM value of 0.7951 and a PSNR of 21.84. Additionally, our approach improves accuracy by 0.2–2.2% over existing baselines on five text recognition datasets, validating the effectiveness of the model.

## 1. Introduction

The task of scene text image super-resolution (STISR) in vision research endeavors to reconstruct high-resolution (HR) textual sequences from their low-resolution (LR) counterparts in scene images. Due to poor imaging conditions, scene text images often suffer from low resolution, significantly hindering text detail acquisition and subsequent tasks such as scene text recognition [1,2,3] and scene text detection [4,5]. Existing methods often leverage text-specific features, such as stroke and character structures, to enhance the clarity and discriminability of super-resolved text images [6,7,8]. This paper focuses on addressing limitations related to the dense distribution of characters and the inefficient representation of foreground sequences amidst complex backgrounds in real scenes.

**Figure 1:**
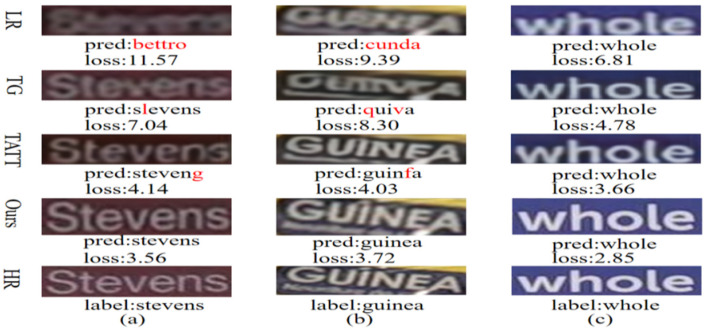
Super-resolution visualization, identification results, and losses of different methods. (**a**) Text-dense regions cause semantic errors in character reconstruction. (**b**) Cluttered backgrounds hinder recognition and complicate super-resolution reconstruction. (**c**) Color drift during reconstruction degrades visual quality.

With the advancement of deep learning, deep convolutional neural networks (DCNNs) have become fundamental to STISR due to their powerful nonlinear mapping capabilities and adaptability. TextSR utilizes a recognition loss to guide the training of generative adversarial networks (GANs). TPGSR [9] integrates semantic features from a text recognizer into its generative network. TATT [10] devised an interpreter and a structural consistency loss to handle text with spatial deformations. As shown in Figure 1a,b, existing methods fail to adequately address the dense character distribution and the adverse impact of complex backgrounds on character representation and reconstruction.

TSRN captures contextual information by integrating sequential residual blocks. TBSRN leverages attention modules to process sequential data, improving robustness for text regions in arbitrary orientations. Gestalt [11] focuses on modeling character stroke-level structures in text images. LEMMA [12] significantly improves the quality of text image recovery by combining local detail enhancement and multi-scale feature fusion. These schemes usually use traditional convolution operations. Their limited receptive fields restrict them to capturing local features such as character strokes and edges. However, as continuous data, the readability of text also depends on global information such as the relative position and overall arrangement of characters. Thus, an effective architecture integrating both local details and global semantics requires further exploration.

Recently, MNTSR [13] employed self-supervised end-to-end memory networks, PerMR [14] fused low-level strokes and high-level semantics from a text recognition network to enhance visual quality, and SGENet [15] incorporated textual semantics with lightweight web design. As shown in Figure 1c, there is often a color drift between the text area and the background area in the images generated by methods similar to the one shown. These methods use semantic information or focus only on the text structure while ignoring the color and texture of the original image.

Aiming at the above bottlenecks, this paper proposes single-character-based embedding feature aggregation using cross-attention for scene text super-resolution tasks, with the following main contributions:

- This study proposes a two-branch feature aggregation strategy, which integrates independently cropped single-character image features with corresponding character probability sequences. This approach ensures that high-level prior information focuses on individual character structures, effectively mitigating complex background interference. It also significantly reduces interference from neighboring densely distributed characters.
- To leverage the complementary capabilities of convolutional kernels with varying receptive fields, an improved inception module is introduced in shallow layers for dynamic multi-scale feature extraction. By dynamically weighting scaled convolutional kernels, the global overview features and fine-grained features are adaptively adjusted for each input, thus enriching the feature expressions to comprehensively understand the salient vision content.
- Leveraging adaptive normalization to learn cross-domain mapping relationships, a color correction operation adaptively adjusts the mean and standard deviation of target images pixels. This enhances super-resolution quality without altering the original image content. Experiments are performed on the public dataset TextZoom, and the results show the superiority of the proposed model compared to the existing baselines. The average recognition accuracy on the test sets of CRNN, MORAN, and ASTER is improved by 1%, 1.5%, and 0.9%, respectively.

## 2. Related Works

### 2.1. Image Super-Resolution

Super-resolution (SR) tasks aim to generate HR images from LR inputs through a prior spatial learning methodology. Traditional SR algorithms include interpolation-based [16,17,18] frequency-domain-based [19,20], and learning-based [21] methods. With the rise of deep learning, Dong et al. [22] pioneered the use of convolutional layers to learn representations for SR reconstruction. Since then, SR networks have been scaled and adapted for STISR tasks, including VDSR [23], an SR architecture using 20 convolutional layers, ESPCN [24], which is formulated using a sub-pixel convolutional network and a recursively structured deep network [25], and a deep residual network [26] with jump connections. SRGAN later introduced adversarial learning for SR reconstruction. Recently, diffusion models have been extended to different vision tasks, including SR after surpassing GANs. For instance, SRDiff [27], the first diffusion-based SR model for single-image reconstruction, effectively addresses spatial detail confusion and training instability to generate realistic results.

### 2.2. Scene Text Recognition for STISR

Scene text recognition (STR) involves automatically detecting and recognizing textual content in natural scenes. Deep learning has driven significant progress in STR. CRNN transforms a text recognition problem into a sequence learning problem, modeling the position and shape of individual characters. MORAN enables text detection and recognition through a spatial transformation network and a recognition network. ASTER [28] introduces an adaptive correction network and an attention-based sequence recognition model. TextSR, TPGSR, TATT, and others leverage these recognizers to extract text prior to guiding SR networks. In this paper, we adopt the above three recognizers to capture semantic information and integrate it with image features for enhanced reconstruction.

### 2.3. Scene Text Super-Resolution

Unlike single-image super-resolution (SISR), STISR aims to enhance text image quality for improved readability and downstream recognition tasks. Although SISR approaches can theoretically be applied to STISR, e.g., Dong et al. performed the STISR task by extending the SRCNN [29] baseline to text images. PlugNet [30] employs a lightweight SR module to extract features from LR images. However, STISR generally requires special processing to maintain the character and clarity of text. Recently, more models specifically designed for STISR have been proposed.

Transformer-based methods (e.g., TATT, C3-STISR [31], and TATSR [32]) leverage the global modeling capabilities of the transformer to capture contextual information in text images, significantly improving restoration performance, which is particularly effective in complex backgrounds and diverse font scenarios. Methods based on diffusion models (e.g., TextDiff [33], RGDiffSR [34], TCDM [35], and PEAN [36]) generate high-quality, high-resolution text images by utilizing diffusion processes, further enhancing image details and visual realism. Semantic and prior-guided approaches (e.g., HiREN [37], PCAN [38], and DPMN [39]) optimize the super-resolution process by incorporating semantic information, text recognition modules, or prior knowledge, significantly improving the semantic restoration quality of text images. These methods demonstrate robust performance in handling low-resolution, blurry, or noise-affected text images.

Furthermore, to address the demands of practical applications, efficient and lightweight methods (e.g., ESTISR [40] and Pixel Adapter [41]) focus on designing computationally efficient super-resolution models suitable for mobile devices and real-time processing scenarios, providing practical technical support for deployment. Notably, the introduction of Real-CE [42] has established a comprehensive dataset and evaluation framework for Chinese–English bilingual scene text image super-resolution, significantly advancing research in multilingual text super-resolution.

## 3. The Proposed Network Architecture

**Figure 2:**
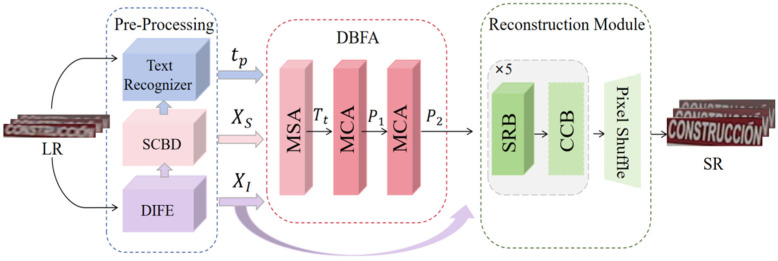
The overall pipeline of the proposed SCE-STISR. LR images are used as input and SR images are used as output. Preprocessing contains text recognizer (TR) single-character boundary detection (SCBD) and dynamic inception feature extraction (DIFE). Dual-branch feature aggregation (DBFA) outputs advanced a prior-guided super-resolution reconstruction.

The proposed SCE-STISR is illustrated in Figure 2. According to it, LR images $X_{LR}$ are used as inputs for preprocessing, which employs DIFE to extract shallow features $X_{I}$ and also takes into account both global and local representations. Then, a pre-trained TR predicts text probability sequences $t_{p}$. A SCBD clip $X_{I}$ is inserted into character feature $X_{S}$ according to character position information. $X_{I}$, $X_{S}$, and $t_{p}$ are parallelly input into DBFA to guide the aggregation of visual and semantic representations. Ultimately, a high-level prior $P_{2}$ = $f_{DBFA}{(X}_{I},X_{S},t_{p})$ is formulated as the input of the reconstruction module, which includes a color correction layer for improving color consistency and accuracy and finally a sequence residual block for performing SR image reconstruction.

### 3.1. Image Preprocessing

The image preprocessing procedure consists of three parts, namely dynamic inception feature extraction, single-character boundary detection, and the text recognizer. Through these steps, LR images $X_{LR}$ are fed as inputs, and shallow features $X_{I}$, character image features $X_{S}$, and text priors $t_{p}$ are extracted as outputs.

#### 3.1.1. Dynamic Inception Feature Extraction

**Figure 3:**
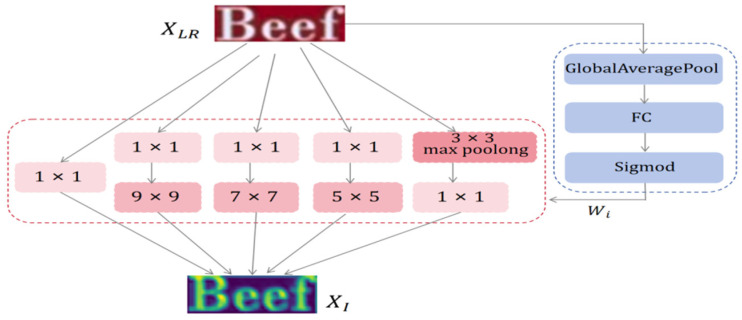
The architecture of DIFE. The red part indicates the multi-scale convolutional kernel extraction for shallow features. The blue portion indicates the computation of the weights of the convolutional kernels corresponding to the input samples.

A single convolutional layer has limited receptive fields, extracting only single-scale features. However, text characters exhibit multi-scale properties, such as font, size, and style, making single convolutional layers inefficient for extracting rich prior features. The DIFE [43] applied in this paper is illustrated in Figure 3. Multi-scale convolution kernels capture structural features at all levels, from letter shapes to word layouts, adapting to diverse datasets and task requirements. A 1 × 1 convolutional kernel reduces or upgrades feature channels, adjusting parameter scales and network nonlinearity for efficient architecture design.

(1) $$X_{i}=f_{c}(k^{h\times h},X_{LR}),h=1,3,5,7,9$$

where $X_{LR}\in R^{H\times W\times 3}$ is the LR text image, where H and W are the height and width, respectively. $X_{i}\in R^{H\times W\times C}$ denotes the shallow features extracted from the text image at each layer with iϵ[0,4], and C denotes the number of feature channels. $k^{h\times h}$ is the size of the convolution kernel sized by h×h, and $f_{c}(\cdot )$ denotes the convolution function.

Since different inputs have different priorities in the feature space captured by multi-scale convolutional kernels, this paper introduces a dynamic feature-weighting mechanism. This mechanism adaptively weights each convolutional kernel branch based on the input feature distribution. This strategy enhances the network’s sensitivity to diverse features and enables it to more accurately understand and improve its ability to process complex text structures, boosting overall performance and generalization. The computational process can be formulated as:

(2) $$w_{i}=f_{sig}\circ f_{FC}\circ f_{GAP}(X_{LR}),i\in [0,4]$$

(3) $$X_{I}=\sum_{i=0}^{4}w_{i}X_{i}$$

where ∘ represents compound operation and $f_{GAP}$ represents the global average pooling operation on input $X_{LR}$. After that, through the $f_{FC}$ fully connected layer function, the respective weight coefficients of the five convolutions of different input samples are learned. Finally, the weights on each dimension are constrained to [0, 1] by sigmoid function $f_{sig}$ to obtain the normalized dynamic weights $w_{i}$. In addition, $X_{I}\in R^{H\times W\times C}$ is the shallow feature output.

#### 3.1.2. Single-Character Boundary Detection

Since LR images usually contain multiple characters, the relative position and order of arrangement between characters are critical. The transformer’s self-attention [44] can directly model the association between any two characters, thereby generating more accurate text position information. To effectively separate foreground text from the background and maximize textual prior information to guide semantic and visual reconstruction, this study adopts transformer-based SCBD [45] to predict the character positions and clip individual characters, forming an image block sequence. 

**Figure 4:**
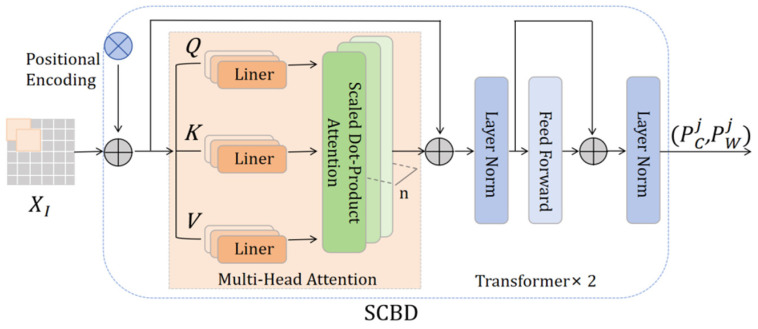
The architecture consists of two transformer encoders with shallow features as inputs and the center coordinates and width of each character as outputs.

SCBD is shown in Figure 4, where the input $X_{I}$ is flattened as $X_{P}=f_{f}(X_{I})$, $X_{P}\in R^{N\times (P^{2}\times C)}$ is a sequence of two-dimensional (2D) image blocks, the resolution of each image block is P×P, and the number of image blocks $N=H\times W/P^{2}$. To help the model maintain position sensitivity while processing the sequence data, a further position coding $E_{pos}$ is fed into the encoder to predict the character position:

(4) $${\left\{(P_{C}^{j},P_{W}^{j})\right\}}_{j=0}^{M}=f_{E}(X_{P}+E_{pos})$$

In Equation (4), $\left(P_{C}^{j},P_{W}^{j}\right)$ refers to the center position and width of each predicted character, j represents the jth character, and $f_{E}(\cdot )$ refers to the cascade transformer encoder.

To reduce background interference, focus on the foreground of the text, and weaken the influence between neighboring characters, we use the predicted positional information $\left(P_{C}^{j},P_{W}^{j}\right)$ to crop out the corresponding single-character features:

(5) $$X_{S}^{j}=f_{clip}(X_{I},(P_{C}^{j},P_{W}^{j}))$$

where $f_{clip}(\cdot )$ denotes the clipping operation, $X_{S}^{j}$ indicates a single-character feature, $X_{S}^{j}$ is flattened as ${\dot{X}}_{S}^{j}=f_{f}(X_{S}^{j}),$ and $X_{S}=({\dot{X}}_{S}^{0},{\dot{X}}_{S}^{1},{\dot{X}}_{S}^{2},...,{\dot{X}}_{S}^{M})$ represents the output of the concatenation of ${\dot{X}}_{S}^{j}$.

#### 3.1.3. Text Recognizer

The aim of the TR is to capture text probability sequences in LR images as prior information, thus guiding the model to reconstruct SR images with precise text semantics. In previous studies, methods such as CRNN, Moran, and Aster show excellent performance in the field of text recognition. Therefore, this paper selects these pre-trained recognition models to obtain the coding vector of the text category $t_{p}^{j}$:

(6) $$t_{p}^{j}=f_{TR}(X_{S}^{j})$$

In this equation, $f_{TR}(\cdot )$ is the TR function and $t_{p}=(t_{p}^{0},t_{p}^{1},t_{p}^{2},...,t_{p}^{M})\in R^{M\times S}$ is the probability sequence of the prior text, where M denotes the length of the sequence, and S is the number of categories in the reference text label set. In general, this set consists of the Arabic numerals 0–9, 26 letters of the alphabet, and a blank character.

### 3.2. Dual-Branch Feature Aggregation

**Figure 5:**
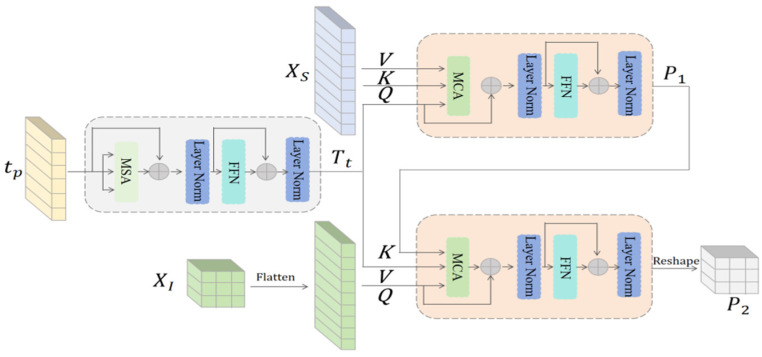
The architecture of DBFA. The whole process is run sequentially from left to right and from top to bottom. The input into the left half is a textual prior, and the output is used as an input to the right cross-attention to guide global and local textual image feature aggregation.

DBFA is a key component in the proposed architecture, as shown in Figure 5. The purpose of DBFA is to interpret prior text $t_{p}$ before image features, thereby exerting precise semantic guidance on relevant spatial locations in the image feature domain. Deep interactions between text priors and image features intensify character adhesion and background interference, causing incorrect semantic guidance. To address these challenges, we focus on aggregating individual character features and global features to supplement missing background information.

(7) $${\bar{T}}_{t}=f_{LN}(f_{MSA}(t_{p})+t_{p})$$

(8) $$T_{t}=f_{LN}(f_{FFN}(T_{t}^{’})+T_{t}^{’})$$

where $f_{LN}(\cdot )$, $f_{MSA}\left(\cdot \right),$ and $f_{FFN}(\cdot )$ refer to functions of the layer norm, multi-head self-attention (MSA), and feedforward network (FFN), respectively. MSA performs global correlations between textual semantic elements and outputs contextually enhanced textual features $T_{t}$.

To achieve depth alignment between text features $T_{t}$ and image features, two-level multi-head cross-attention (MCA) is used, where text feature $T_{t}$ and single-character feature $X_{S}$ are used as first-level MCA inputs, with $T_{t}$ as the query and $X_{S}$ as the key and value, allowing each character to find the image feature that corresponds to it. The input tensors $X_{S}$ and $T_{t}$ are first split into n sub-tensors in the channel dimension.

(9) $$Q_{i}=T_{t}W_{i}^{Q},K_{i}=X_{S}W_{i}^{K},V_{i}=X_{S}W_{i}^{V},\;\;\;i=1,2,...,n$$

where $W_{i}^{Q}$, $W_{i}^{K}$, and $W_{i}^{V}$ are the linear mapping matrices corresponding to the ith attention head, respectively.

(10) $$P_{1}^{i}=softmax(\frac{Q_{i}{K_{i}}^{T}}{\sqrt{d_{k}}})V_{i}$$

In this equation, $P_{1}^{i}$ is the calculated attention per head and $d_{k}$ is the length of $K_{i}$ for scaling the attention. We process the results $P_{1}^{i}$ with a channel-wise concatenation operation $f_{con}$(·) and a linear projection function $f_{w}$, described as:

(11) $${\bar{P}}_{1}=f_{w}\circ f_{con}(P_{1}^{1},P_{1}^{2},...,P_{1}^{n})$$

The output ${\bar{P}}_{1}$ is passed to a FFN for feature refinement, obtaining a high-level prior $P_{1}$ without background interference.

In addition, the prior $P_{1}$ contains character image block $X_{S}$ as a key, text features $T_{t}$ as a value, and the size is consistent. It can be directly input into the secondary MCA to avoid dimensional inconsistencies. We use $P_{1}$ to connect semantic information and global visual features. We flatten the shallow features $X_{I}$ as the query. Unlike the $X_{S}$ in first-level MCA, $X_{I}$ is not flattened according to single characters, and the background and foreground information is not clear in terms of the primary and secondary. However, $X_{I}$ contains global features, which effectively improves the visual quality problem caused by missing backgrounds. The $P_{1}$ is a key and $T_{t}$ is a value.

(12) $${\bar{P}}_{2}=f_{LN}(f_{MCA}(X_{I},P_{1},t_{p})+X_{I})$$

(13) $$P_{2}=f_{LN}(f_{FFN}({\bar{P}}_{2})+{\bar{P}}_{2})$$

Each pixel point in $X_{I}$ can be queried by $P_{1}$ to the text feature corresponding to it, obtaining a high-level prior $P_{2}$ for valid semantic mappings.

Since the cropped image is a sequence of image block features arranged by character, cluttered background interference is avoided when using transformer aggregation. When calculating the correlation, this reduces the interference of the neighboring characters on the recovery of the current character. Global features $P_{2}$ can more accurately assign semantic information to the spatial domain via $P_{1}$.

### 3.3. Reconstructed Module

The reconstruction module in this study consists of a sequential residual block (SRB), a color correction block (CCB), and an upsampling layer. $P_{2}+X_{I}$ is sent to the SRB for SR reconstruction. Subsequently, the resulting features are again sent to the SRB for element-by-element summation with $X_{I}$ to ultimately obtain the SR reconstruction features $X_{SR}^{C}$. However, the SR image restored through upsampling the reconstructed features $X_{SR}^{C}$ via the pixel-shuffle layer may suffer from color drifting in either the text part or the background part. In view of this, this study considers adaptive normalization to adjust the feature representation of the image to match the statistical properties of the target domain. This ensures that the model can generate images consistent with the target style.

In detail, the mean and variance of the color values of the SR image and LR image were first calculated using the $f_{M}(\cdot )$ and $f_{V}(\cdot )$ functions as the target criterion:

(14) $$\mu_{X_{I}}^{C},\mu_{X_{SR}}^{C}=f_{M}(X_{SR}^{C},X_{I}^{C})$$

(15) $$\sigma_{X_{I}}^{C},\sigma_{X_{SR}}^{C}=f_{V}(X_{SR}^{C},X_{I}^{C})$$

where c denotes the RGB channel and $\mu_{X_{SR}}^{C}$,$\mu_{X_{I}}^{C}$ and $\sigma_{X_{SR}}^{C}$,$\sigma_{X_{I}}^{C}$ denote the mean and variance for $X_{SR}^{C}$ and $X_{I}$. Secondly, $X_{SR}^{C}$ is normalized to improve the model’s accuracy:

(16) $$X_{SR}^{N}=\frac{X_{SR}^{C}-\mu_{X_{SR}}^{C}}{\sigma_{X_{SR}}^{C}}$$

Subsequently, by applying $\mu_{X_{I}}^{C}$ and $\sigma_{X_{I}}^{C}$ to the normalized feature $X_{SR}^{N}$, the SR image is adjusted so that its color value’s mean and variance are consistent with those of the LR image to obtain the color-corrected reconstructed feature ${\bar{X}}_{SR}$.

(17) $${\bar{X}}_{SR}=X_{SR}^{N}\cdot \sigma_{X_{I}}^{C}+\mu_{X_{I}}^{C}$$

The reconstruction process in this study consists of five SRB and CCB modules connected in series, with a final upsampling layer to obtain the reconstructed high-resolution image features $X_{SR}$.

### 3.4. Loss Function

In this study, the total loss function consists of pixel loss $L_{pix}$ and text recognition prior loss $L_{tp}$.

(18) $$L_{pix}={\left\|X_{SR}-X_{HR}\right\|}_{2}$$

The $L_{pix}$ loss generates high-quality images from LR images by constraining the $L_{2}$ paradigm of super-resolution images and high-resolution images.

(19) $$L_{tp}=\beta {\left\|A_{SR}-A_{HR}\right\|}_{1}+\gamma KL(t_{p},t_{HR})$$

In Equation (19), β and γ denote hyperparameters, $A_{HR}$ is the distribution of attention that the model expects to see, and $A_{SR}$ is the distribution of attention that the model actually sees. $A_{HR},A_{SR}\in R^{B\times S\times (H\times W)}$, B is the batch size, and S is the maximum length of the text. $A_{HR},A_{SR}$ are obtained when the multi-head attention mechanism calculates the alignment relationship between text and image features. $t_{p}$ and $t_{HR}$ denote the text probability sequences of SR and HR images obtained by the text recognizer. $L_{tp}$ denotes that the text recognition branch is fine-tuned by constraining the $L_{1}$ paradigm and the Kullback–Leibler divergence of the text prior recognized from the LR image and the real image. The total loss function L is expressed as follows.

(20) $$L=L_{pix}+\alpha L_{tp}$$

In Equation (20), α is the equilibrium parameter. During the training process, the loss function is used as the optimization objective, and this error signal is then fed to the network through the back-propagation iterations to update all the module parameters.

## 4. Experimental Results and Discussion

According to this study, DBFA, as the main component based on cross-attention, captures deep interactions between global and local feature semantics for visual reconstruction. In addition, DIFE dynamically adjusts the multi-scale feature weights according to the different structures, shapes, and distributions of the inputs. Moreover, the CCB adaptively relieves the color distortion caused by background interference. After the experimental configurations are presented, as seen in Section 4.1, the effectiveness of these proposed components is verified by ablation experiments in Section 4.2. In Section 4.3, we further evaluate the model’s performance on TextZoom through comparative tests and demonstrate its robustness on the STR dataset.

### 4.1. Dataset and Experimental Details

TextZoom: This STISR dataset is derived from two state-of-the-art SISR datasets, RealSR and SRRAW, captured by a multifocal camera in the field. These datasets are more realistic and challenging than synthetic data. TextZoom uses a field-of-view matching and transforming approach to initially align the images with different focal lengths and crop the text images with the same-sized text box to obtain textual images with different resolutions. Textual images with larger focal lengths serve as HR images, while those with smaller focal lengths serve as LR images. Smaller focal lengths result in blurrier image details. According to stratified random sampling based on scene types, 17,367 pairs of LR–HR training sets and 4373 pairs of test sets are included in TextZoom, with text annotations, border types, and raw focal lengths. The test set is divided into three subsets: 1619 samples for the easy subset, 1411 for the medium subset, and 1343 for the hard subset.

STR dataset: To evaluate effectiveness across different data distributions, five English STR datasets—ICDAR2013 [46], ICDAT2015 [47], SVT [48], SVTP [49], and CUTE80 [50]—are used. Specifically, ICDAR2013 contains 1015 test samples, while ICDAR2015 contains 2077 samples. The text in these images may appear in diverse scenes, exhibiting issues such as distortion, occlusion, and uniform illumination. SVT contains 350 test samples with significant scale variations and complex backgrounds. The text may be bent or distorted, and the varying lighting conditions increase recognition difficulty. SVTP contains 645 test samples. Although the data are synthetic, the text images are high-quality but lack the complexity of real scenes. CUTE80 contains 288 text images with characters arranged along curved paths, forming curved text lines. These images have high resolution and quality but no LR counterparts. In preprocessing, the images with resolutions smaller than 16×64 are selected and degraded with Real-ESRGAN [51] to test the robustness of the model.

The STISR benchmarks are implemented using the PyTorch 1.13.1 framework, and all experiments are performed on a single RTX4090 GPU. The number of MSA mechanisms in SCBD is set to four and the number of SRBs is set to five. In addition, the model with an input batch size of 64, image width of 64, and height of 16 is optimized using Adam [52]. The number of training rounds is set to 500 and the learning rate is set to 0.001, which yields an output with a width of 128 and a height of 32. In this study, three text recognizers, ASTER, CRNN, and MORAN, are applied to assess the recognition accuracy. To assess the quality of image reconstruction, we adopt the peak signal/noise ratio (PSNR) and structural similarity index measure (SSIM) [53] as reference metrics.

### 4.2. Ablation Experiment

In this section, the effectiveness of the components DIFE, DBFA, and CCB are validated on TextZoom, with CRNN applied as a text recognizer.

#### 4.2.1. The Role of Dual-Branch Feature Aggregation

DBFA is designed to accurately interpret the semantic information $t_{p}$ and the corresponding positions in the image features $X_{I}$, and align between the textual prior and the image features. Then, it is compared with other textual prior interpreters: firstly, $t_{p}$ is fused with $X_{I}$ using the inverse convolution block. Secondly, $t_{p}$ and $X_{I}$ are aligned using the SFT layer [54] to obtain parameter pairs based on a few textual prior conditions. Then, an affine transformation is applied to adaptively fuse each intermediate feature map. Finally, TPI is used to compute the correlation between the textual prior and image features to guide the SR textual reconstruction. To verify the effectiveness of DBFA, local text feature aggregation (LTFA) is separately tested as the textual prior interpreter.

**Figure 6:**
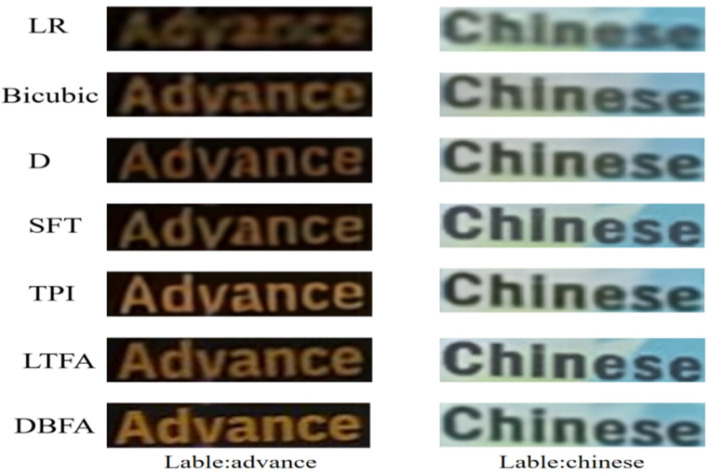
Visualization of super-resolution reconstructed images using different decoders.

Table 1 shows that the DBFA component achieves the highest recognition accuracy and average accuracy across the easy, medium, and hard subsets. Additionally, the PSNR and SSIM metrics are optimal, demonstrating excellent SR performance. As shown by $f_{D}$ (50.6%) and $f_{SFT}$ (49.2%), although textual priors guide the reconstruction, they are not accurately assigned to corresponding positions in the image space, leading to the underutilization of prior information. Notably, TPI (52.8%) aligns image features with the text regions effectively but fails to distinguish the foreground and background clearly, and the attention mechanism does not sufficiently allocate weights to the text regions. Using only local textual feature integration (53.1%) ignores the background’s influence and reduces the visual quality. In contrast, DBFA focuses on both the foreground and background, accurately aligning text priors with image features. This improves recognition accuracy to 53.6%, PSNR to 21.84, and SSIM to 0.7997, validating its effective super-resolution reconstruction performance. The visual comparison in Figure 6 shows that the text image obtained by the proposed method has the best visual quality.

#### 4.2.2. The Role of Dynamic Inception Feature Extraction

In this study, DIFE is mainly applied to multi-scale feature extraction, similar to the work in [32]. Small convolutional kernels (e.g., 1 × 1, 3 × 3) capture fine details such as edges and textures, making them suitable for extracting low-level features. Medium convolutional kernels (e.g., 5 × 5, 7 × 7) have a moderate receptive field, capturing both local and partial global information, and are suitable for extracting medium-level features such as object shapes or silhouettes. Large convolutional kernels (e.g., 9 × 9) have a large receptive field, making them suitable for extracting high-level features such as the overall shape of objects or scene context. The dynamic weighting strategy adaptively adjusts the proportion of multi-scale feature information extracted from different convolutional kernels according to different input distributions. In this experiment, we analyze the relationship between convolutional kernel sizes and the inclusion of the dynamic weighting strategy, evaluating their impact on feature extraction and recognition accuracy, as shown in Table 2.

**Figure 7:**
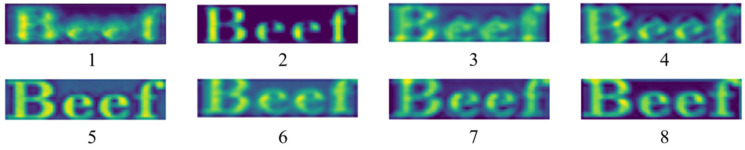
Text image shallow-feature extraction map. Numbers 1–8 correspond to the eight sets of comparative experimental feature visualization results in Table 2, respectively.

In this table, the combination of multi-scale convolutional kernels demonstrates significant advantages, outperforming single convolutional layer feature extraction. Small convolution kernels ignore contextual information to reduce accuracy, while large convolution kernels do not capture enough details. Experimental results indicate that DIFE achieves the best recognition accuracy (53.6%) at the eighth set of convolution kernel sizes. The visualization in Figure 7 denotes that the module is highlighted in the character region, with the best retention of global and detailed features.

#### 4.2.3. Validity of the CCB Module

**Figure 8:**
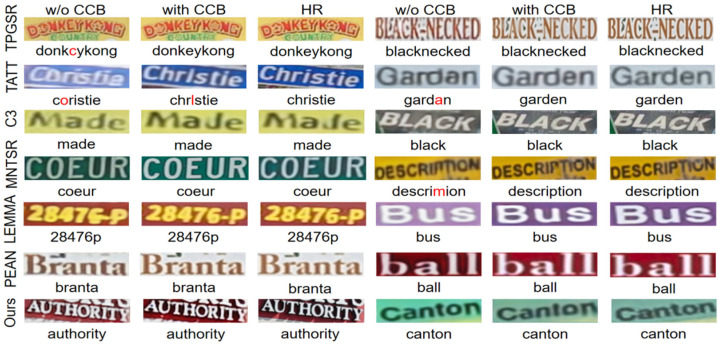
Visualization of super-resolution results with and without CCB incorporation for different models.

To evaluate the effectiveness of the CCB in color correction, five models—TPGSR, TATT, C3-STISR, MNTSR, LEMMA, PEAN, and SCE-STISR—were selected to compare results with and without the CCB module. Table 3 shows that all models improved their text recognition accuracy or SR quality, which indicates that this color correction module effectively improves the image reconstruction quality. Specifically, our model SCE-STISR improves the recognition accuracy from 53.9% to 55.3% and the average recognition accuracy from 53.0% to 53.6% on the medium difficulty subset after using CCB, while PSNR and SSIM improve from 21.43 and 0.7982 to 21.84 and 0.7997, respectively, which demonstrates a significant performance improvement. In contrast, other models such as LEMMA and PEAN also show some improvement in PSNR and SSIM after using the CCB, although the improvement in recognition accuracy is smaller. We also provide some qualitative comparative studies by comparing the baseline model with the SR image visualization after adding the CCB. As shown in Figure 8, we find that existing methods suffer from color defects, while our method constrains the images to maintain color consistency, resulting in higher image and visual quality.

#### 4.2.4. Effectiveness and Efficiency of Different Components

As shown in Table 4, the impact of different module combinations on model performance is evaluated. The experiment denotes that the model’s recognition accuracy is optimal (53.6%) when three modules—DBFA, DIFA, and CCB—are integrated simultaneously. Although the performance improvement with these modules resulted in a slight decrease in inference speed (↓10%) and an increase in the number of parameters (↑66.7%), the recognition accuracy was significantly improved (↑1.5%). Given the compact input size of the STISR model, the existing architecture already excels in inference speed and parameter efficiency. Therefore, a moderate increase in complexity to improve SR performance is justified.

### 4.3. Comparison with State-of-the-Art Results

#### 4.3.1. TextZoom Quantitative Research

To validate the model, the proposed SCE-STISR was compared with SISR methods (e.g., SRCNN, SRResNet, EDSR, RCAN, CARN, and HAN) and STISR models (e.g., TSRN, TBSRN, PCAN, TPGSR, Text Gestalt, TATT, C3-STISR, MNTSR, PerMR, TEAN, DPMN, LEMMA, PEAN, and TCDM).

Table 5 shows the recognition accuracy metrics based on the CRNN, MORAN, and ASTER recognizers on the TextZoom test set. Since SISR methods are designed for generalized SR, they do not account for the unique character structures or textual information in text images. The results show that the recognition accuracy of SISR methods is generally low. Most of the other STISR methods use a single convolution layer, which is unable to take into account both character details and textual context and treats the foreground and background equally. Compared with the STISR baseline TATT, we improve the average recognition accuracy of CRNN, MORAN, and ASTER by 1%, 1.4%, and 0.9%, respectively, demonstrating the effectiveness of the method. Although the TCDM and PEAN models demonstrate excellent performance in terms of average recognition accuracy for the CRNN, MORAN, and ASTER recognizers, the experimental details mentioned in the literature—such as batch size, graphics card performance, and other parameters—reveal that the characteristics of diffusion models result in high computational complexity, time-consuming training, and slow inference. This makes it challenging to run these models in real time on resource-constrained devices. Additionally, the multi-step iterative generation process of diffusion models imposes strict requirements on the quality and scale of training data, which inevitably increases training costs and the risk of data dependency. These factors collectively limit the deployment and widespread adoption of TCDM and PEAN in practical application scenarios.

The results of SSIM and PSNR, which are common evaluation metrics for SR, are shown in Table 6. Our method effectively constrains the super-resolution reconstructed image to be infinitely close to the low-resolution image in the color channel by introducing the CCB (color consistency block), thereby ensuring the consistency of the output image with the input image in terms of statistical features. This design not only significantly improves the visual quality of the image but also enhances structural similarity, addressing the issues of color drift and low reconstructed visual quality caused by the neglect of overall image quality in previous methods. Compared to existing methods, our model achieves the best PSNR on the difficult subset (20.78) and the simple subset (24.99), as well as the best average PSNR (21.84). Additionally, it achieves the best performance on the medium subset (0.6955) and the difficult subset (0.7859), as well as the best average SSIM (0.7951), which fully validates the effectiveness of the model in enhancing image quality. These results demonstrate that our innovative approach has significant advantages in improving image structural similarity and color consistency. Although the character recognition accuracy is not yet optimal, our superior performance in terms of the PSNR and SSIM metrics proves the success of the model design in improving overall image quality. This provides higher-quality inputs for downstream text recognition tasks and offers important optimization directions for subsequent research.

To evaluate the statistical significance of the model outputs, five existing benchmarks were tested. Table 7 demonstrates the average accuracy, PSNR, and SSIM metrics for each method on the test set, along with their corresponding *p*-values. The results show that the proposed model outperforms the existing benchmarks in most of the metrics and exhibits more stable performance (smaller variance) over multiple training sessions. The significance test results (*p*-values) further confirm that our method has a statistically significant improvement compared to existing methods. Based on these quantitative results, the SCE-STISR model significantly outperforms other benchmarks in terms of generation quality.

#### 4.3.2. TextZoom Qualitative Research

**Figure 9:**
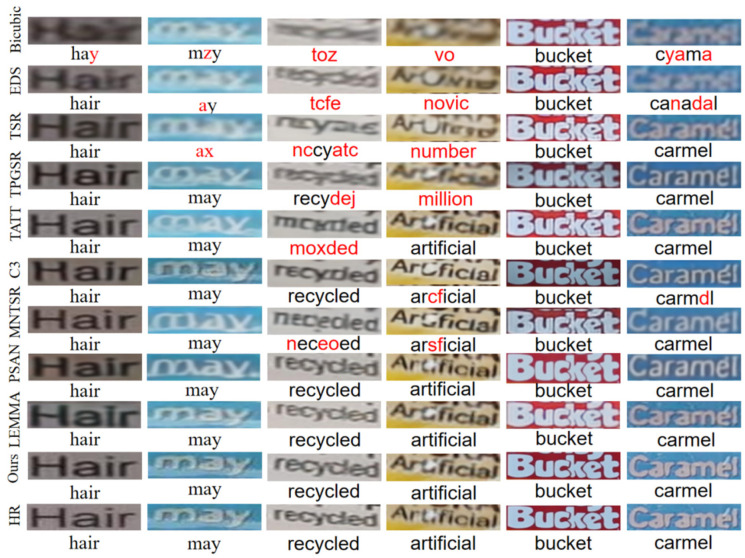
Visualization of reconstruction results on the TextZoom dataset.

As shown in Figure 9, to further validate the model’s effectiveness, the visualization results of image reconstruction are presented under different configurations, including text lengths, backgrounds, colors, and text recognition results.

The observations show that all methods outperform the bicubic interpolation method, but there is a significant difference in visual quality between SISR and STISR. The text-specific model SCE-STISR achieves the best visualization results, which are closest to the HR images. As shown in the first and second columns of the figure, although most methods can accurately recognize characters, issues such as blurred character structures and bend strokes may occur during text SR reconstruction. In addition, SCE-STISR accurately assigns semantic information to corresponding image features, reconstructs images correctly, and achieves satisfactory visual results. In addition, the model performs well in dealing with the dense connection of characters and the background influence on foreground restoration, such as ‘recycled’ and ‘artificial’, producing reconstruction results that are clear and the closest to the high-resolution image. In contrast, the other methods show the phenomenon of character misconstruction. In previous methods, the color of characters or the background is easily inconsistent with the color of LR and HR images, resulting in large errors, such as ‘bucket’ and ‘caramel’, while this method effectively corrects the color drift and significantly improves the fidelity of images.

#### 4.3.3. Quantitative Research of Text Recognition Datasets

To further verify its generalization ability, the proposed model is tested on five STR datasets, ICDAR2013, ICDAR2015, SVT, SVTP, and CUTE80, using the parameters trained on TextZoom. These datasets are primarily derived from real-world scenes and contain text of varying lengths and complex backgrounds. LR images are selected to form the test set, and due to the high quality of most of the images, the Real-ESRGAN second-order degradation method is used to degrade the quality. As shown in Table 8, SCE-STISR aligns text priors with images more accurately, provides precise guidance, and achieves better results.

#### 4.3.4. Research on Densely Connected Datasets

**Figure 10:**
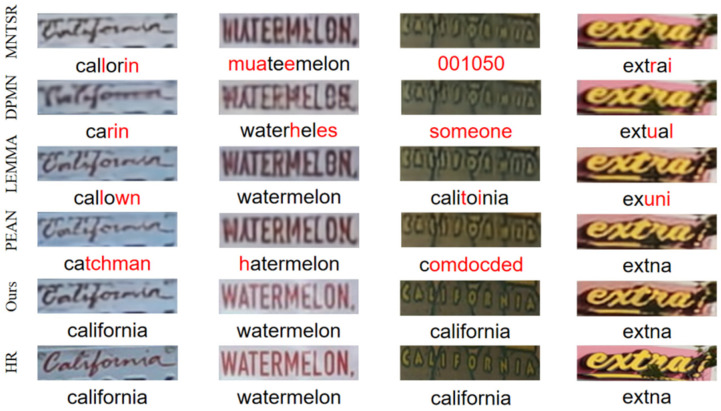
The left side of the figure shows the SR results for text-dense connected images, and the right side shows the SR results for complex background text images.

Since our model aims to mitigate the effects of complex background interference and dense text connectivity, this section compares the text recognition accuracies and image qualities of different methods on datasets featuring complex backgrounds and densely connected text. The experiments are conducted on 650 text images with complex backgrounds or densely connected characters, selected from TextZoom, ICDAR2013, ICDAR2015, SVT, SVTP, CUTE80, and COCO-Text and all with a resolution lower than 16 × 64. As shown in Table 9, our model achieves a recognition accuracy of 42.7% on the CRNN recognizer, outperforming MNTSR (38.9%), DPMN (35.4%), LEMMA (40.8%), and PEAN (39.5%). On the MORAN and ASTER recognizers, SCE-STISR achieves recognition accuracies of 54.1% and 57.3%, respectively, surpassing all other models. This performance is primarily attributed to the BDFA module, which effectively mitigates interference between complex backgrounds and adjacent characters, improves the accuracy of character separation and localization, and enhances image quality by optimizing color distribution. These innovations enable SCE-STISR to exhibit enhanced robustness in handling densely connected text and complex backgrounds. The visualization results in Figure 10 further demonstrate the advantages of our model in recovering image details.

#### 4.3.5. Robustness Test

This section aims to evaluate the model’s robustness according to common image degradations. Experiments are conducted on 500 randomly selected samples from the STR dataset and simulate real scenarios through two levels of degradation processing: firstly, a Gaussian fuzzy kernel is applied (kernel widths of 1, 3, and 5, respectively), followed by the introduction of additive Gaussian noise (covariance values are set to 10, 30, and 50). A CRNN is used as the text recognizer to compare the baseline TATT with related methods. As shown in Table 10, the performance advantage becomes more pronounced as blur and noise intensity increase, particularly under extreme conditions (kernel width of 5, covariance of 50), where recognition accuracy improvements remain significant. These results show that the proposed model can be efficiently generalized to out-of-distribution datasets while exhibiting stronger robustness to unknown degradation types.

#### 4.3.6. Discussion

**Figure 11:**
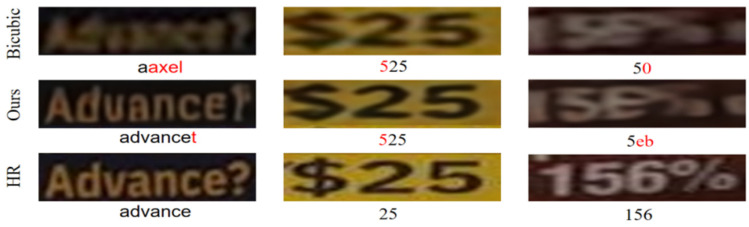
Text recognition visualization results for unknown characters in the text label character set.

Although the proposed SCE-STISR architecture can efficiently generate English characters and numbers, a few limitations still remain. Firstly, its recognition accuracy on the SVT dataset is only 15.1%. This dataset is characterized by drastic changes in sample scale, curved text morphology, and curved character arrangement, which pose a serious challenge to the recognition ability of the model. In addition, our model might focus too much on the extraction of local character features, thus ignoring the spatial relationship between characters to some extent. In addition, the model is highly dependent on the distribution of the training data. As shown in Figure 11, when encountering languages, characters, or symbols that are not present in the training data, the recognizer may misrecognize them, resulting in confusing results. Furthermore, the introduction of DBFA increases computational complexity and reduces inference speed. Although the feature extraction mechanism of the character recognizer differs somewhat from the design goal of our module, resulting in the recognition accuracy not surpassing some state-of-the-art algorithms, this does not diminish the innovation and effectiveness of our approach. Our core goal is to mitigate the image quality problems associated with background interference, character tightness, and color drift. Future work may focus on designing extended architectures that do not require textual prior guidance and exploring ways to balance reconstruction quality with inference speed.

## 5. Conclusions

In this study, we propose single-character embedding feature aggregation based on cross-attention for scene text super-resolution. To address the limitation of single convolution layers in capturing only single-scale features, we employ DIFE for multi-scale feature extraction and dynamically adjust feature maps across different receptive fields based on input variations. In addition, we used DBFA, which distinguishes the textual part from the background part by a single character and then uses a semantic prior to guide the image. Finally, a CCB is implemented to improve the color drift problem in the reconstruction process by normalizing the color channel. In this study, ablation experiments are conducted for DIFE, DBFA, and the CCB to validate the effectiveness of each model component. To further demonstrate the superiority of this study, we conducted qualitative and quantitative comparison experiments on TextZoom using 16 benchmarks, as well as quantitative evaluations on five STR datasets and text-dense connected datasets. Without a significant increase in the number of model parameters, our approach achieves superior results in both text recognition accuracy and image quality assessment metrics.

## Figures and Tables

**Table 1:** Comparing several text–image feature aggregation modules, where $f_{D}$ denotes the deconvolution operation, $f_{SFT}$ is the SFT layer fusion operation, and TPI is the TP interpreter in TATT. LTFA indicates that only local features are used in DBFA to guide cross-attention.↑ indicates that the higher the performance of the metric, the better it is, and blackened indicates the optimal metric.

Fusion Strategy	Easy	Medium	Hard	avgAcc ↑	PSNR ↑	SSIM ↑
w/o DBFA	51.2%	41.9%	31.7%	41.6%	21.02	0.7690
$f_{D}$	61.8%	52.1%	37.9%	50.6%	21.10	0.7819
$f_{SFT}$	60.3%	50.4%	36.9%	49.2%	20.87	0.7783
TPI	62.9%	53.5%	39.8%	52.8%	21.52	0.7930
LTFA	63.1%	**53.8%**	39.8%	53.1%	21.43	0.7954
DBFA	**63.5%**	55.3%	**39.9%**	**53.6%**	**21.84**	**0.7997**

**Table 2:** Effect of different convolutional kernel sizes on recognition accuracy on the TextZoom dataset. Here, the dynamic indicates that a dynamic weighting mechanism is performed.

	DIFE Parameter	Easy	Medium	Hard	avgAcc
1	9 × 9	62.8%	53.6%	38.7%	52.6%
2	1 × 1, 1 × 1 + 5 × 5	62.4%	52.1%	38.6%	52.5%
3	1 × 1, 1 × 1 + 7 × 7	63.2%	53.7%	38.9%	52.7%
4	1 × 1, 1 × 1 + 9 × 9	63.4%	53.9%	39.1%	52.9%
5	1 × 1, 1 × 1 + 3 × 3, 7 × 7 + 1 × 1	62.9%	53.5%	39.5%	53.4%
6	1 × 1, 1 × 1 + 9 × 9, 5 × 5 + 1 × 1	63.6%	54.6%	39.7%	53.2%
7	1 × 1, 1 × 1 + 5 × 5, 1 × 1 + 7 × 7, 1 × 1 + 9 × 9, 3 × 3 + 1 × 1	63.8%	54.8%	39.8%	53.4%
8	1 × 1, 1 × 1 + 5 × 5, 1 × 1 + 7 × 7, 1 × 1 + 9 × 9, 3 × 3 + 1 × 1 (dynamic)	63.5%	55.3%	39.9%	53.6%

**Table 3:** Effect of different convolutional kernel sizes on recognition accuracy on the TextZoom dataset. Here, √ indicates the addition of the CCB, and × indicates not adding the CCB, and blackened indicates the optimal metric.

Approach	CCB	Easy	Medium	Hard	avgAcc	PSNR	SSIM
TPGSR	×	61.0%	49.9%	**36.7%**	49.8%	21.02	0.7690
	√	**62.1%**	**51.6%**	**36.7%**	**50.4%**	**21.32**	**0.7705**
TATT	×	**62.6%**	53.4%	**39.8%**	52.6%	**21.52**	0.7930
	√	62.4%	**54.4%**	39.6%	**52.7%**	20.95	**0.7951**
C3-STISR	×	**65.2%**	53.6%	**39.8%**	53.7%	**21.51**	0.7721
	√	65.1%	**54.0%**	39.6%	**53.8%**	21.37	**0.7853**
MNTSR	×	**64.3%**	54.5%	38.7%	**53.3%**	21.53	0.7946
	√	64.0%	**54.8%**	**38.9%**	53.2%	**21.67**	**0.7964**
SCE-STISR	×	63.3%	53.9%	39.8%	53.0%	21.43	0.7982
	√	**63.5%**	**55.3%**	**39.9%**	**53.6%**	**21.84**	**0.7997**
LEMMA	×	67.1%	58.8%	40.6%	56.3%	21.43	0.7543
	√	**67.2%**	58.6%	**40.8%**	**56.4%**	**21.59**	**0.7623**
PEAN	×	**68.9%**	60.2%	45.9%	59.0%	21.57	0.7946
	√	68.8%	**60.3%**	**46.0%**	**59.1%**	**21.78**	**0.8017**

**Table 4:** Effect of combining different modules. TPI is used instead when the DBFA module is not included. CRNN is used to recognize the text in SR images. Frames per second (FPS) is used to evaluate the speed of model inference. Parameters are used to measure model size. √ means join the module, and blackened indicates the optimal metric.

DBFA	DIFA	CCB	Recognition Accuracy				FPS	Parameters
			Easy	Medium	Hard	avgAcc		
-	-	-	62.26%	52.73%	39.09%	52.1%	44	30.5 M
√	-	-	62.94%	52.73%	39.46%	52.4%	40	50.5 M
√	√	-	63.25%	53.93%	39.76%	53.0%	39	50.9 M
√	√	√	**63.53%**	**55.31%**	**39.95%**	**53.6%**	39	51.2 M

**Table 5:** Quantitative comparison of SCE-STISR and previous state-of-the-art methods using three recognizers, CRNN, MORAN, and ASTER. The higher the recognition accuracy, the better the text super-resolution. Boldface represents the optimal result, strikethrough represents the sub-optimal result, and avg represents the average recognition result. _ indicates a sub-optimal indicator, and blackened indicates the optimal metric.

Method	CRNN				MORAN				ASTER			
	Easy (%)	Medium (%)	Hard (%)	Avg (%)	Easy (%)	Medium (%)	Hard (%)	Avg (%)	Easy (%)	Medium (%)	Hard (%)	Avg (%)
Bicubic	36.4	21.1	21.1	26.8	60.6	37.9	30.8	44.1	67.4	42.4	31.2	48.2
SRCNN	41.1	22.3	22.0	29.2	63.9	40.0	29.4	45.6	70.6	44.0	31.5	50.0
SRResNet	45.2	32.6	25.5	35.1	66.0	47.1	33.4	49.9	69.4	50.5	35.7	53.0
EDSR	42.7	29.3	24.1	32.7	63.6	45.4	32.2	48.1	72.3	48.6	34.3	53.0
RCAN	46.8	27.9	26.5	34.5	63.1	42.9	33.6	47.5	67.3	46.6	35.1	50.7
CARN	40.7	27.4	24.3	31.4	58.8	42.3	31.1	45.0	62.3	44.7	31.5	47.1
HAN	51.6	35.8	29.0	39.6	67.4	48.5	35.4	51.5	71.1	52.8	39.0	55.3
TSRN	52.5	38.3	31.4	41.4	70.1	55.3	37.9	55.4	75.1	56.3	40.1	58.3
PCAN	59.6	45.4	34.8	47.4	73.7	57.6	41.0	58.5	77.5	60.7	43.1	61.5
TBSRN	59.6	47.1	35.3	48.1	74.1	57.0	40.8	58.4	75.7	59.9	41.6	60.1
Gestalt	61.2	47.6	35.5	48.9	75.8	57.8	41.4	59.4	77.9	60.2	42.4	61.3
TPGSR	63.1	52.0	38.6	51.8	74.9	60.5	44.1	60.5	78.9	62.7	44.5	62.8
TATT	62.6	53.4	39.8	52.6	72.5	60.2	43.1	59.5	78.9	63.4	45.4	63.6
C3-STISR	65.2	53.6	39.8	53.7	74.2	61.0	43.2	59.5	79.1	63.3	46.8	64.1
PerMR	65.1	50.4	37.8	52.0	76.7	58.9	42.9	60.6	80.8	62.9	45.5	64.2
MNTSR	64.3	54.5	38.7	53.3	76.7	61.2	44.9	61.9	79.5	64.6	45.8	64.4
TEAN	63.7	52.5	38.1	52.2	76.8	60.8	43.4	61.4	80.4	64.5	45.6	64.6
DPMN	64.3	54.1	39.2	53.3	73.2	61.4	43.8	60.4	79.2	64.0	45.0	63.8
TCDM	67.3	57.3	42.7	55.7	77.6	62.9	45.9	62.2	81.3	65.1	50.1	65.5
PEAN	**68.9**	**60.2**	**45.9**	**59.0**	**79.4**	**67.0**	**49.1**	**66.1**	**84.5**	**71.4**	**52.9**	**70.6**
LEMMA	67.1	58.8	40.6	56.3	77.7	64.4	44.6	63.2	81.1	66.3	47.4	66.0
SCE-STISR	63.5	55.3	39.9	53.6	73.9	59.5	44.7	60.9	80.9	63.4	45.8	64.5

**Table 6:** Quantitative comparisons of the CRNN between SCE-STISR and previous state-of-the-art methods show that the higher the PSNR and SSIM, the better the quality of the text super-resolution image, _ indicates a sub-optimal indicator, and blackened indicates the optimal metric.

Method	PSNR				SSIM			
	Easy	Medium	Hard	Avg	Easy	Medium	Hard	Avg
Bicubic	22.35	18.98	19.39	20.35	0.7884	0.6254	0.6592	0.6961
SRCNN	23.48	19.06	19.34	20.78	0.8379	0.6323	0.6791	0.7227
SRResNet	24.36	18.88	19.29	21.03	0.8681	0.6406	0.6911	0.7403
EDSR	24.26	18.63	19.14	20.68	0.8633	0.6440	0.7108	0.7394
RCAN	22.15	18.81	19.83	20.26	0.8525	0.6465	0.7227	0.7406
CARN	22.70	19.15	20.02	20.62	0.8384	0.6412	0.7172	0.7323
HAN	23.30	19.02	20.16	20.95	0.8691	0.6537	0.7387	0.7596
TSRN	25.07	18.86	19.71	21.42	0.8897	0.6676	0.7302	0.7690
PCAN	24.57	19.14	20.26	21.49	0.8830	0.6781	0.7475	0.7752
TBSRN	23.46	19.17	19.68	20.91	0.8729	0.6455	0.7452	0.7603
Gestalt	23.95	18.58	19.74	20.76	0.8611	0.6621	0.7520	0.7584
TPGSR	23.73	18.68	20.06	20.97	0.8805	0.6738	0.7440	0.7719
TATT	24.72	19.02	20.31	21.52	0.9006	0.6911	0.7703	0.7930
C3-STISR	24.71	19.03	20.09	21.51	0.8545	0.6674	0.7639	0.7721
PerMR	24.89	18.98	20.42	21.43	0.9102	0.6921	0.7658	0.7894
MNTSR	24.93	19.28	20.38	21.50	**0.9173**	0.6860	0.7806	0.7946
TEAN	-	-	-	21.70	-	-	-	0.7850
DPMN	24.84	19.08	20.51	21.49	0.9013	0.6902	0.7695	0.7925
LEMMA	24.67	19.21	20.37	21.43	0.8734	0.6783	0.5601	0.7543
PEAN	24.89	**19.46**	20.41	21.75	0.9157	0.6901	0.7837	0.7946
SCE-STISR	**24.99**	19.13	**20.78**	**21.84**	0.9038	**0.6955**	**0.7859**	**0.7951**

**Table 7:** Statistical tests of different methods. We compute the mean accuracy, PSNR, and SSIM after five rounds of training with different models and the corresponding *p*-values. *p*-values are obtained by our method and other methods in turn.

Method		SCE-STISR	TPGSR	TATT	C3-STISR	MNTSR
PSNR	mean	21.84	20.95	21.51	21.51	21.52
	*p*-value	-	3.7 × 10^−6^	4.5 × 10^−5^	9.1 × 10^−5^	2.8 × 10^−3^
SSIM	mean	0.7951	0.7719	0.7940	0.7716	0.7941
	*p*-value	-	1.4 × 10^−5^	1.4 × 10^−1^	2.3 × 10^−5^	6.7 × 10^−1^
avgAcc	mean	53.6	51.9	52.6	53.7	53.4
	*p*-value	-	1.5 × 10^−4^	2.8 × 10^−4^	1.6 × 10^−2^	1.8 × 10^−2^

**Table 8:** Quantitative research on five text recognition datasets including IC13, IC15, CUTE80, SVT, and SVTP. Here, CRNN is used to recognize the text in SR images. Blackened indicates the optimal metric.

Method	STR Datasets				
	IC13	IC15	CUTE80	SVT	SVTP
Bicubic	9.6%	10.1%	35.8%	3.3%	10.2%
SRResNet	11.4%	13.4%	50.5%	9.3%	13.8%
TSRN	15.6%	18.6%	66.9%	10.0%	16.4%
TBSRN	17.7%	21.3%	75.0%	12.2%	17.4%
TPGSR	22.7%	24.2%	72.6%	13.7%	16.5%
TATT	27.6%	28.6%	74.7%	14.2%	25.9%
C3-STISR	24.7%	22.7%	71.5%	10.2%	17.7%
SCE-STISR	**28.9%**	**30.7%**	**74.9%**	**15.1%**	**26.5%**

**Table 9:** Comparing the text recognition accuracy of different methods on densely connected datasets. Blackened indicates the optimal metric.

Method	CRNN	MORAN	ASTER
MNTSR	38.9%	49.3%	52.0%
DPMN	35.4%	46.2%	49.6%
LEMMA	40.8%	53.3%	55.7%
PEAN	39.5%	52.4%	54.8%
SCE-STISR	**42.7%**	**54.1%**	**57.3%**

**Table 10:** Results of degraded images with Gaussian noise using Gaussian fuzzy kernels with different kernel widths and different covariances. Blackened indicates the optimal metric.

Kernel Width r	Method	σ=10	σ=30	σ=50
r = 1	TATT	58.1%	51.4%	47.3%
	Ours	**58.8%**	**52.9%**	**49.2%**
r = 3	TATT	47.4%	42.8%	37.7%
	Ours	**48.5%**	**44.7%**	**40.1%**
r = 5	TATT	39.8%	35.5%	31.6%
	Ours	**41.3%**	**37.5%**	**34.7%**

## Data Availability

Data are contained within the article.
